# Analysis of short-term functional outcomes of colorectal resections in older adults aged 75 years and older: a prospective single health board cohort study

**DOI:** 10.1308/rcsann.2025.0042

**Published:** 2025-06-19

**Authors:** V May, C MacKay, G Ramsay

**Affiliations:** ^1^ NHS Grampian, UK; ^2^ Aberdeen Centre for Evaluation, University of Aberdeen, UK

**Keywords:** Older adults, Colorectal, Quality of life, Functional recovery, Mortality

## Abstract

**Introduction:**

The global increase in life expectancy is likely to lead to a higher number of older patients undergoing colorectal resections. This is an understudied cohort, with available data concentrating on generic surgical outcomes such as postoperative complications and mortality rates. Few studies have explored quality of life and return to baseline in this cohort.

**Methods:**

Inpatient outcome data on all patients aged 75 years and over who underwent colorectal resection in our region between 2018 and 2023 were collected prospectively. Patient characteristics, complication rates, functional decline and mortality data were documented. These data were supplemented with a subsequent review of death, readmission, and admission to a non-own home environment post-surgery.

**Results:**

Of 408 colorectal surgery patients, 303 were elective and 105 were emergency cases. Of these cases, 59.4% (*n* = 180) of elective cases and 71.4% (*n* = 75) of emergency patients experienced a postoperative complication. In total, 35.5% of patients experienced a functional decline with reduced mobility or ability to perform independent care. Emergency patients (*n* = 21, 20%) demonstrated a higher mortality rate at 1 year than elective cases (*n* = 25, 8.3%).

**Conclusion:**

Colorectal surgery in patients aged 75 years and older presents significant risks, particularly in emergencies. This study documents high rates of functional decline, complications and mortality in elderly patients. It highlights the need for individualised surgical care planning and enhanced perioperative counselling, and helps quantify this functional deterioration.

## Introduction

Approximately 40% of colorectal cancers diagnosed in the United Kingdom (UK) are in patients aged over 75 years (State of the Nation Report, National Bowel Cancer Audit).^1^ Furthermore, Scandinavian data have shown that the incidence of older colorectal cancer has increased, and the median age of patients is now between 72 and 74 years.^2–5^ Therefore, the management of cancer in older patients is now a common part of colorectal practice. Although the aim of colorectal resection in both cancer and benign conditions remains curative and prolongation of life, older patients have a higher perioperative mortality rate, and survival may be affected by other comorbidities. Interestingly, some work in this patient group has demonstrated that there is a higher priority for quality of life and functional independence than long-term survival.^6^ How our treatment affects functional independence is an understudied question.

Existing research focuses on generic surgical outcomes, such as mortality, morbidity and length of hospital stay. There are fewer assessments of geriatric-focused events or return to pretreatment levels of functional attainment. It is also unclear how many patients require long-term care following surgery.^2,6^ Although treatment strategy should be decided on a case-by-case basis and tailored to the individual needs of the patient, for some patients with potentially operable colorectal disease, the proposed intervention (surgery) will be inferior to symptom control and no operation.^7^

In older patients, perceived treatment benefits and potential adverse effects should be considered carefully given comorbidities, frailty and baseline function. In people aged over 80 undergoing colorectal cancer surgery, functional decline is seen to be one of the most important outcomes to consider by the patients themselves, because it significantly affects the post-surgical quality of life.^6^ There is therefore somewhat of a disconnect between what is valued by the patient and what is studied by the clinical community.

The Emergency Laparotomy in Frailty study demonstrated a strong link between frailty and post-surgical outcomes.^8,9^ However, this work explored emergency presentations in which interventions occur in a time-critical manner with less capacity for discussion around functional baseline. There is little assessment of similar effects in the elective sphere. Such data would serve as guidance for patient counselling, facilitating informed patient-centred decision-making by elucidating potential declines in quality of life, function and independent care. Having estimations of such events would help patients in the assessment of risks and benefits of the procedure. It would also be useful data in the consideration and discussion of treatment escalation or advanced care planning before such interventions occur. Estimations of likelihood of deteriorations in functional status, as well as the duration of time to recovery will be helpful for clinicians, because they would help guide discussions and facilitate the delivery of individualised care.

In this study, we therefore seek to assess how older patients recover after colorectal surgery. Our aim was to analyse both standard surgical outcomes and functional recovery and discharge to care facilities in patients aged 75 years and older undergoing colorectal surgery.

## Methods

### Study design

This is a prospective single-centre cohort study conducted at Aberdeen Royal Infirmary, a tertiary centre serving a population of approximately 600,000 people spread over 3,000 square miles in North-East Scotland, Orkney and Shetland.^10,11^

### Patient population

Patients were identified on the local prospectively collected database of all individuals having major operations in the colorectal team in the unit. There were no data available on those patients who did not have an operation. All patients undergoing colorectal resection aged 75 years or older were included. Elective and emergency cases were included for both minimally invasive (robotic or laparoscopic) and open surgical approaches. Exclusion criteria were defined as patients younger than 75 years and those who did not undergo surgical management.

### Data collection

Data on every patient undergoing a colorectal resection were collected prospectively over 5 years from June 2018 to September 2023. Data collected included age, comorbidities, surgical approach, complication severity (Clavien–Dindo classification), mortality and functional decline (mobility and care requirements post-surgery). Functional decline was defined as a postoperative reduction in preadmission functional baseline of mobility, dependency on care or both. Data relating to functional decline were retrieved from clinical notes written by other members of the multidisciplinary team, including occupational therapists, physiotherapists and nursing staff. A patient was deemed to have had a reduction in their functional baseline if their status of mobility or care dependency had declined from admission data to the discharge location on their formal discharge letter.

Outcomes and accuracy of data were reviewed weekly by consultants and senior change nurses in a data meeting.

### Data analysis

Data were analysed using Microsoft Excel. Patient data were divided into two cohorts: elective and emergency surgeries, with subgroup analysis on functional recovery and long-term post-surgery outcomes. Categorical data were analysed using a chi-squared test. Continuous data (such as length of hospital stay) were reported as the median and interquartile range (IQR).

### Registration

This study was registered with the NHS Grampian Quality Improvement and Assurance (QIA) team under project ID 6059 and approved as an evaluation of current practice.

## Results

### Patient characteristics

A total of 408 patients were included in this study. These were subdivided into 303 elective surgeries (median age: 80 years, range: 75–90 years) and 105 emergency surgeries (median age = 80.5 years, range: 75–91 years). In elective cases, 155 (51.2%) were female and 148 (48.8%) were male, whereas for emergency presentations, 59 (56.2%) were female and 46 (43.8%) were male. Emergency patients had a significantly higher median American Society of Anesthesiologists physical status grade (median 3 in emergencies, 2 in elective cases, *p* < 0.001). The most common comorbidities were hypertension (56.8% in elective cases, 45.7% in emergencies), gastro-oesophageal reflux disease (45.5% in elective cases, 8.6% in emergencies) and osteoarthritis (18.8% in elective cases, 21.9% in emergencies). Malignancy was the most common diagnosis for elective operations (78.5%). Emergency surgeries were most frequently due to bowel obstruction (60%) and bowel perforation (14.3%); only 5.7% of emergency surgeries were due to malignancy without either of the above complications (Table 1).

**Table 1:** Patient characteristics

	**Elective cohort (*n* = 303)**	**Emergency cohort (*n* = 105)**	***p-*value**
Median age (range)	80 (75–90)	80.5 (75–91)	0.09
Sex, *n* (%)			
Male	148 (48.8)	46 (43.8)	
Female	155 (51.2)	59 (56.2)	
Median no. of comorbidities (range)	3 (0–15)	4 (0–12)	0.16
Diagnosis, *n* (%)			
Malignancy	238 (78.5)	6 (5.7)	
Polyp	41 (13.5)	0 (0)	
Prolapse	14 (4.6)	0 (0)	
Obstruction	1 (0.4)	63 (60)	
Perforation	0 (0)	15 (14.3)	
Bleeding	0 (0)	4 (3.8)	
Other	9 (3)	17 (16.2)	
Median ASA grade (range)	2 (1–4)	3 (1–5)	<0.001*
Surgical approach, *n* (%)			
Open	150 (49.5)	93 (88.6)	
Minimally invasive	153 (50.5)	12 (11.4)	
Type of operation performed, *n* (%)			
Right hemicolectomy	147 (48.5)	23 (21.9)	<0.001*
Defunctioning stoma formation	28 (9.2)	23 (21.9)	<0.001*
Hartmann’s procedure	19 (6.3)	24 (22.9)	<0.001*
Anterior resection	17 (5.6)	1 (1)	0.008*
APER	16 (5.3)	0	<0.001*
Subtotal colectomy	6 (2)	10 (9.5)	<0.001*
TAMIS	26 (8.6)	0	0.196
Small bowel resection	2 (0.7)	14 (13.3)	<0.001*
Other	42 (13.9)	10 (9.5)	0.196
Stoma formation	116 (38.3)	116 (38.3)	<0.001*

APER = Abdomino Perineal Excision of Rectum; ASA = American Society of Anesthesiologists (ASA) physical status classification system; TAMIS = Transanal minimally invasive surgery; * denotes statistical significance

### Perioperative approach

Minimally invasive surgical approaches were used more frequently in elective presentations (50.5%) than in emergencies (11.4%). The rate of stoma formation was high in both cohorts. However, emergency patients had a significantly higher formation rate when compared with elective cases (79% in emergencies, 38.3% in elective cases, *p* < 0.001; Table 1).

**Table 2:** Short-term postoperative outcomes

	**Elective cohort (*n *= 303)**	**Emergency cohort (*n *= 105)**	***p-*value**
Postoperative location, *n* (%)			
Ward	173 (57.1)	23 (21.9)	<0.001*
HDU	118 (38.9)	74 (70.5)	<0.001*
ITU	4 (1.3)	6 (5.7)	0.03*
Unknown	8 (2.6)	2 (1.9)	0.54
Clavien–Dindo Classification Scale, *n* (%)			
0	123 (40.6)	30 (28.6)	0.02*
I	82 (27.1)	21 (20)	0.14
II	84 (27.7)	32 (30.5)	0.7
IIIa	1 (0.3)	4 (3.8)	0.07
IIIb	5 (1.7)	3 (2.9)	0.58
IV	4 (1.3)	8 (7.6)	0.01*
V	4 (1.3)	7 (6.7)	0.02*
Median length of stay, days (IQR)	7 (0–38)	12 (1–85)	<0.001*
Medically fit for discharge, *n* (%)	291 (96)	86 (81.9)	<0.001*
Died in hospital, *n* (%)	4 (1.3)	7 (6.7)	0.03*
Discharge location, *n* (%)			
Home	274 (90.4)	79 (75.2)	0.001*
Rehabilitation facility	7 (2.3)	11 (11.6)	0.01*
Nursing home	9 (3)	4 (3.8)	0.08
Sheltered housing	8 (2.3)	3 (2.9)	0.91
Palliative care	1 (0.35)	1 (1)	0.55

HDU = high-dependency unit; IQR = interquartile range; ITU = intensive treatment unit; * denotes statistical significance

### Complications rates and time to discharge

The overall complication rate was high in both groups. However, elective patients were significantly less likely to experience surgical complications than emergency patients, as 40.6% of elective cases experienced no complications compared with 28.6% for emergency presentations (*p* = 0.02). Elective patients were also significantly less likely to experience more serious complications, as only 4.6% of elective patients experienced a complication score >3 on the Clavien–Dindo classification scale, compared with 21% in the emergency cohort (*p* < 0.001). Elective cases were significantly more likely to return to ward-level care, which includes advanced observations beds (57.1% in elective cases, 21.9% in emergencies, *p* < 0.001) following their procedure. The emergency cohort was also more likely to require a higher level of care postoperatively in the high-dependency unit (70.5% in emergencies, 38.9% in elective cases, *p* < 0.001). Table 2 illustrates the complication rates across the two cohorts.

The length of stay from surgical procedure to discharge was analysed (Table 2). Overall, emergency patients required significantly longer hospital stays (median 12 days, IQR 8–20) than elective cases (median 7 days, IQR 5–11, *p* < 0.001).

### Functional decline

Overall, 35.5% of patients (32.7% elective, 43.8% emergencies) experienced a post-surgical functional decline at the point of discharge with a substantial documented reduction in mobility and independent care (Table 3). As a direct result of this, emergency presentations were more likely to be discharged to a non-own home environment following their procedure (18% of emergencies and 4% of elective cases). Many of these patients required admission to a rehabilitation or long-term care facility (Table 2).

### Readmission rates

There was no significant difference in the median number of readmissions to hospital between the cohorts (both groups had a median of one readmission). However, it was notable that the emergency group experienced a wider range in the number of readmissions (0–19 readmissions in emergencies, 0–8 readmissions in elective cases). The most frequent locations of readmission are illustrated in Table 4. Emergencies had a significantly higher rate of readmission to general medicine (32.6% in emergencies, 19.8% in elective cases, *p* = 0.004). There was no significant difference observed in rates of readmission to other specialities (Table 4).

**Table 3:** Functional decline

	**Elective cohort (*n *= 303)**	**Emergency cohort (*n *= 105)**	***p-*value**
Reduction in functional baseline	99 (32.7)	46 (43.8)	0.05
Care	23 (23.2)	11 (23.9)	0.9
Mobility	49 (49.5)	21 (45.7)	0.74
Both	27 (27.3)	14 (30.4)	0.72

Values are given as *n* (%)

**Table 4:** Readmission

	**Elective cohort (*n *= 303)**	**Emergency cohort (*n *= 105)**	***p-*value**
Median no. of readmissions (range)	1 (0–8)	1 (0–19)	0.4
No. of patients readmitted (%)	158 (52.1)	55 (52.4)	1
No. of patients not readmitted (%)	145 (47.9)	50 (47.6)	1
Location of readmission, *n* (%)			
General surgery	100 (29.7)	41 (29.1)	0.83
General medicine	66 (19.8)	46 (32.6)	0.004*
Geriatrics	37 (11.1)	10 (7.1)	0.15
Others	131 (39.4)	44 (31.2)	0.09

* denotes statistical significance

**Table 5:** Mortality

	**Elective cohort (*n *= 303)**	**Emergency cohort (*n *= 105)**	***p-*value**
No. of deaths (%)	59 (19.5)	34 (32.4)	0.01*
Median age of death (range)	84 (75–91)	83 (75–90)	0.45
Median time from operation to death, days (IQR)	490 (0–1,672)	231.5 (1–1,660)	<0.001*
Cause of death, *n* (%)			
Metastatic colorectal cancer	19 (32.2)	11 (32.4)	1
Colorectal cancer	13 (22)	4 (11.8)	0.21
Unknown	8 (13.6)	11 (32.4)	0.06
Other	19 (32.2)	8 (23.4)	0.41

IQR = interquartile range; * denotes statistical significance

### Mortality

**Figure 1:**
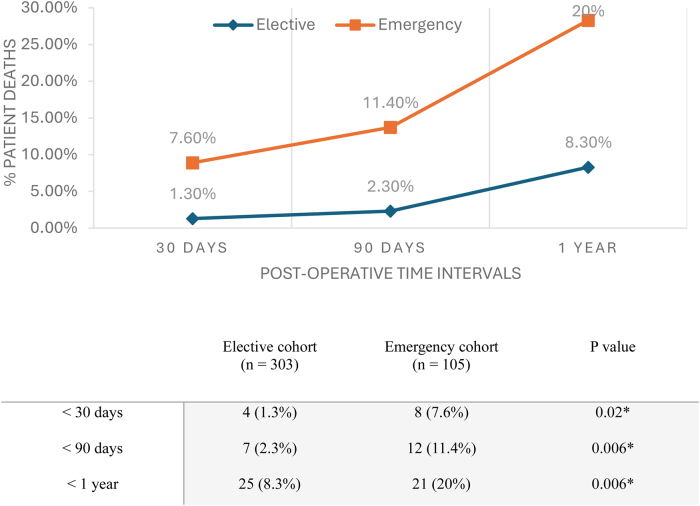
Mortality within postoperative time intervals (* denotes statistical significance)

The in-hospital mortality rates were 6.7% in emergencies and 1.3% in elective cases (*p* = 0.02, Table 2). The total number of patients that had died at the end of the censor was 93 (22.8%,Table 5). The median time from procedure to death was significantly shorter in emergency presentations (231.5 days in emergencies, 490 for elective cases, *p* < 0.001). Mortality was examined at specific postoperative time intervals; overall, emergency patients see a higher mortality rate at each time interval compared with elective patients (Figure 1). A significantly higher mortality rate was observed at all postoperative time intervals, particularly at 1-year post-surgery (8.3% in elective cases, 20% in emergency, *p* = 0.006).

## Discussion

In this study, we quantify the high-risk profile for the population of older patients undergoing colorectal resectional surgery. Patients aged 75 years and older had a 35.5% rate of functional decline, with 32.7% (*n* = 99) of elective patients and 43.8% (*n* = 46) of emergencies being discharged with reduced mobility, increased care needs or both. Older patients in this age group also had an overall inpatient complication rate of 62.5%, with a total of 7.6% deemed medically unfit for discharge, and the overall one-year mortality rate was 11.3%. To our knowledge, this study is the first assessment of functional recovery in an elective and emergency UK colorectal cohort of older patients.

There is no universal definition of the ‘older’ patient. This is dependent on cultural environments and deprivation, and can be country specific.^12–14^ We adopted a pragmatic decision to include all patients aged 75 and over. This age threshold is also used by the National Bowel Cancer Audit (NBOCAP) and is helpful for comparing data.^15^ One notable finding is that most of this cohort had few significant comorbidities. The most common were hypertension, osteoarthritis and gastric reflux. Thus, this appears to be a cohort of patients that include those that are ‘good for their age’; nevertheless, the complication profile, functional decline and mortality rates were substantial. However, our high stoma rates would infer that the operating surgeons perceive their risks to be high and have aimed to reduce these by avoiding anastomoses.

It is perhaps intuitive that some older patients undergoing colorectal resection commonly prioritise quality of life over long-term survival. The functional recovery of patients post-surgery, particularly in terms of returning to independent living, is an important outcome for patients. It is therefore one that should be monitored more by colorectal clinicians. Our findings align with previous studies in different environments, in describing the significant proportion of patients who experience functional decline after colorectal surgery.^10^ The reduction in functional capacity is particularly relevant given that it often leads to delayed discharge or transfer to long-term care facilities. Early mobilisation and rehabilitation post-surgery aim to avoid this functional decline, especially in elective cases where prehabilitation may offer benefits.^4^ Such data are important for preoperative counselling of patients.

The long inpatient stay and high readmission rate per patient is striking. It is noticeable that some patients are admitted on multiple occasions to several different specialities. This is important when resource management is being considered. Furthermore, these patients may be able to be predicted as potentially delayed discharges, which can cause considerable challenges to the National Health Service care structure. Should patients with reduced independence and functional decline be able to be predicted, their care assessments and needs could be changed pre-emptively in the prehabilitation phase of their clinical management. Care should also be taken in interpreting the readmission data because these could be associated with events unrelated to the operation. A risk stratification of functional decline or a predictive model for patients likely to be readmitted is beyond the scope of this study. Should this be developed, it may be a crucial adjunct to the preoperative conversations and assist in shared decision-making for such individuals.

There is increasing evidence that a patient’s chronological age may not be an accurate prognostic indicator for postoperative outcomes.^16–19^ Rather, it is important to consider the patient’s functional ability and underlying health conditions. There are few studies exploring comorbidities in the older population undergoing colorectal surgery. We demonstrate that the risks are high for all older patients and suspect that the physiological insult of the operation may unmask frailty in those individuals hitherto not diagnosed with other significant comorbidities. Franklyn *et al* emphasised the use of nonoperative management in the older patient population with colorectal malignancy while also considering underlying comorbidities.^20^ In their cohort of patients aged 75 years and older, they identified a 586-day median life expectancy from diagnosis; this is higher than the median life expectancy we observed in both the elective (490 days) and emergency (231 days) presentations.

However, our unit lacked regular preoperative assessment by geriatricians during this study period. Such data highlight the importance of a careful, patient-centric multi-speciality review before patients decide to embark on such procedures. This is not unusual because there appears to be a general paucity of geriatric pathways that concentrate on the specific problems faced by older surgical patients in the UK, despite evidence that such approaches can improve outcomes.^21,22^

### Study strengths

The use of data produced from a prospectively maintained database with careful reporting of complications is an advantage because the accuracy of the data was reviewed weekly by senior members of both the medical and nursing teams. Notably, our complication rates are high across both the elective and emergency groups. Our overall complication rate of 62.5% in this cohort contrasts with those observed by previous studies, which reported 21%, 30.3% and 35%.^23–25^ After careful and extensive review, we believe that this does not reflect a poor standard of clinical care, but is reflective of our prospective complication data collection. Our accurate weekly documentation of all the complications that occur in hospital reflects an honest assessment of our outcomes and we suspect that this will reflect similar outcomes elsewhere in the country. This is emphasised by highlighting that the mortality results in this study reflect a similar elective 1-year mortality rate of 8.3% to that reported in NBOCAP of 7.5%.^15^ Furthermore, our 30-day mortality rate is 2.9%, which is significantly lower than rates reported by previous studies at 12%.^26^

### Study limitations

However, this study is not without limitations. The size of the cohort in the current study was relatively low at only 408 patients. We think this is still an important addition to the literature because it demonstrates the potential scale of the problem and is likely to be generalisable to other UK centres. Assessing reduction in the functional baseline as a patient outcome carries some challenges. The data relating to the decline in function were extrapolated from notes by other members of the multidisciplinary team (occupational therapists, physiotherapists and nursing staff), and therefore the number of patients who experienced a functional decline may be underestimated because of variations in reporting. We also did not have regular geriatrician input pre- or postoperatively during the timeframe of this study. Furthermore, we had no access to data on the group of individuals for whom an operation was potentially possible, but not undertaken (the NoLap patients). This might explain our right-sided predominance in this cohort.^27^ Finally, our prehabilitation has been undertaken on an informal basis. Although access to physical optimisation was available, it is unclear as to how many patients opted for this support.

## Conclusion

Colorectal surgery in patients aged 75 years and older presents significant risks, particularly in the emergency setting. Data collected by this study over 5 years highlight the high rates of functional decline, complications and mortality in this cohort, underscoring the necessity for individualised surgical pathways and enhanced perioperative counselling. Although not often on the colorectal surgical radar, such geriatric outcome assessments will be an important part of surgery in the 21st century owing to changes in national demographics.
